# P-53 - AUTOMATED DETECTION OF INVASIVE PNEUMOCOCCAL DISEASE CLUSTERS IN CLOSED SETTINGS: PILOT IMPLEMENTATION IN THE SOUTH WEST AND IMPLICATIONS FOR NATIONAL ROLL OUT

**DOI:** 10.1093/pubmed/fdag046.086

**Published:** 2026-07-09

**Authors:** Ms Sarah Leaver, Dr Joshua Khan, Dr Alasdair Wood, Dr Ellen Heinsbroek, Dr Nicholas Young, Dr Claire Brown

**Affiliations:** UK Health Security Agency; UK Health Security Agency; UK Health Security Agency; UK Health Security Agency; UK Health Security Agency; UK Health Security Agency

## Abstract

**Background:**

Invasive pneumococcal disease (IPD) poses a substantial risk in closed settings such as care homes, where vulnerable individuals are at risk of rapid transmission and severe disease, including septicaemia, pneumonia and meningitis. Early detection of IPD clusters supports timely intervention, including infection control, chemoprophylaxis and vaccination to limit spread and reduce morbidity.

**Objectives:**

To describe the implementation and evaluation of a regional pilot of an automated IPD cluster detection tool in the South West of England, compare its performance with the previous manual process, and consider the implications for a national roll-out.

**Methods:**

Laboratory-confirmed IPD results reported to the UKHSA laboratory surveillance system are automatically ingested into the UKHSA Case and Incident Management System (CIMS). Previously, new IPD cases were manually reviewed to identify epidemiological links. In July 2025, the UKHSA South West team introduced an automated weekly process to identify IPD clusters in closed settings. This process uses an R script to extract laboratory-confirmed IPD reports from CIMS and identify potential clusters: two or more cases occurring at the same postcode within 14 days of the date entered. The tool was assessed for feasibility and reliability and compared with the manual method to evaluate improvements in operational efficiency, sensitivity and public health value.

**Results:**

Between July and December 2025, the tool detected two IPD clusters in separate care homes, leading to prompt antibiotic chemoprophylaxis and vaccination of eligible residents. Evaluation indicated that the automated tool was reliable, straightforward to use and provided meaningful time-saving benefits compared to the manual process. The once-weekly schedule of the process was highlighted as a limitation.

**Conclusions:**

The pilot demonstrates that automated IPD cluster detection facilitates cluster identification and supports timely public health response within closed settings. Further refinement, particularly regarding frequency of the process, will support effective national roll-out and further strengthen IPD surveillance across England.

